# The use of ondansetron for the treatment of nausea in dogs with vestibular syndrome

**DOI:** 10.1186/s12917-021-02931-9

**Published:** 2021-06-21

**Authors:** S. Foth, S. Meller, H. Kenward, J. Elliott, L. Pelligand, H. A. Volk

**Affiliations:** 1 Department of Small Animal Medicine and Surgery, University of Veterinary Medicine Hannover, Hannover, Germany; 2 Department of Clinical Science and Services, Royal Veterinary College, Hertfordshire Hatfield, UK; 3 Department of Comparative Biomedical Sciences, Royal Veterinary College, Hertfordshire Hatfield, UK

**Keywords:** Vestibular syndrome, Ondansetron, Nausea, Behavioural Assessment

## Abstract

**Background:**

Vestibular syndrome is often accompanied by nausea. Drugs currently approved for its treatment have been developed to stop vomiting but not nausea. The efficacy of 5-HT_3_ receptor antagonists to reduce nausea has been described for chemotherapy, but not for nausea secondary to vestibular disorders.

**Methods:**

Sixteen dogs with vestibular syndrome-associated nausea were included in the open-label, multicentre study. The intensity of nausea-like behaviour was analysed before ondansetron administration (0.5 mg/kg i.v.) and 2 h afterwards, using a validated 5-point-scale. The occurrence and frequency of salivation, lip licking, restlessness, vocalisation, lethargy, and vomiting were assessed.

**Results:**

All dogs initially showed signs of nausea, whereas only 31% showed vomitus. The intensity of nausea was significantly reduced in all dogs (*p* ≤ 0.0001) 2 h after ondansetron administration, including the clinical signs of nausea analysed in 11 dogs (salivation [*p* = 0.0078], lip licking [*p* = 0.0078], restlessness [*p* = 0.0039], and lethargy [*p* = 0.0078]) except for vocalisation (*p* > 0.9999).

**Conclusions:**

The results provide preliminary evidence of the potential benefit of ondansetron in the treatment of nausea, which was present in all examined dogs. Vomiting was only observed in 5 dogs indicating that nausea can occur separately and should not be perceived only as a preceding stimulation of the vomiting centre.

**Supplementary Information:**

The online version contains supplementary material available at 10.1186/s12917-021-02931-9.

## Background

Dogs with dysfunctions of the vestibular system are frequently encountered in primary veterinary care [1], with an overall prevalence of 0.08% in primary care and an even higher prevalence of 0.36% in dogs aged 9 years or older [2]. Dysfunctions of the vestibular system do manifest in various neurological clinical signs, such as head tilt, nystagmus, or ataxia [3, 4]. Additionally, signs of kinetosis (motion sickness) can also occur due to the fact that the nucleus of the solitary tract, which is responsible for nausea and emesis, receives vestibular input [5]. Thompson et al. (2009) [6] pointed out that various common central and peripheral vestibular diseases in humans (e.g., peripheral and central vestibular syndrome, Meniere’s syndrome, vestibular neuritis, labyrinthitis and vestibular migraine) are associated with symptoms of nausea and vomiting and require intervention with anti-nausea medication. Due to similar pathways in the central nervous system in both species, it can be assumed that dogs perceive nausea as individually and diversely as human patients do [7, 8]. Nausea is a complex and multi-dimensional sensation. It is not only affected by vestibular inputs but also by many other factors like somatic stress, stimuli from the gastro-intestinal tract, and by physical and psychological factors [9].

The sensation of nausea has a protective function and is often associated with the urge to vomit. However, nausea is not implicitly associated with the result of vomiting. Nausea can be perceived in different intensities and be associated with a preceding stimulation of the vomiting centre, which leads to vomiting only at higher intensity. Nevertheless, the incorrect assumption that dogs without vomiting are consistently not nauseous is omnipresent in veterinary practice. Nausea is pharmacologically much more difficult to control than vomiting. This is why Horn et al. (2007) [10] assumed that the neurobiological systems that cause vomiting and nausea are at least partially separated [10].

The currently approved medications in veterinary medicine for the treatment of nausea and vomiting in cats and dogs – e.g., maropitant (neurokinin-1 receptor antagonist) and metoclopramide (dopamine D2 receptor antagonist) – successfully limit vomiting. Yet dogs and other animals still show clinical signs of nausea after their use. Studies confirm that maropitant and metoclopramide do not have a clinically relevant effect against nausea compared to placebo [11–20]. Interestingly, 5-HT_3_ receptor antagonists, such as ondansetron, can eliminate both nausea and vomiting [8, 13, 15]. Ondansetron is a selective 5-HT_3_ receptor antagonist, whose central effect may be explained by the high distribution rate of 5-HT_3_ receptors in the area postrema, which is associated with the nucleus of the solitary tract and by the pivotal role of the central serotonergic (5-HT) system for both nausea and vomiting [21, 22]. Furthermore, a large proportion of 5-HT_3_ receptors was detected in the periphery (vagal nerve, enteric neuronal elements of the gastrointestinal tract), whose activation, accompanied by an increase of vasopressin levels, culminates in gastric dysrhythmias [21, 23]. Ondansetron has already been proven as a successful treatment of chemotherapy-related nausea and vomiting in both human and animals, and showed clear advantages over antidopaminergic drugs (e.g., metoclopramide), antihistamines, neurokinin-1-receptor antagonists (e.g., maropitant) and anticholinergics [24]. Similar results have been documented in experiments with ferrets [25] and further experiments have shown ondansetron being superior to other antiemetics in cases of nausea due to reasons other than a vestibular disturbance [13, 15, 23, 26].

In general, the clinical efficacy assessment of anti-nausea drugs is difficult [27]. Detecting and grading the subjective status of nausea is a requirement in drug development. Evaluating nausea is strongly linked to precise observations of facial expressions and behaviour. Typical pathophysiological and behavioural patterns such as salivation, licking lips, restlessness, lethargy, and vocalisation can be witnessed [17]. The appearance of these prodromal signs and autonomic responses has a higher probability by a rising intensity of nausea [28]. Due to the lack of communication capabilities, the detection of nausea in dogs is even more complicated compared to humans. Furthermore, dogs may mask signs during their visit to the clinic because of stress and discomfort, so that symptoms are even more subtle. A numerical rating scale (NRS) established by Rau et al. (2010) [17] and further developed by Kenward et al. (2015) [29], which evaluates the general nausea and the prodromal signs on a 5-point-scale, supports precise and conscious behavioural assessment. The NRS acts as an additional tool for observers to reliably detect even mild clinical signs of nausea.

Studies addressing the issue of treatment and assessment of nausea due to vestibular disorders have not yet been published to the authors’ knowledge. Veterinary experience has been limited to studying nausea and vomiting induced by chemotherapeutic and other drugs [8, 12, 18, 20, 25]. Therefore, the aim of this case series is to describe the clinical experience of treatment of nausea resulting from vestibular syndrome in dogs with ondansetron, a 5-HT_3_ receptor antagonist, evaluated by behavioural assessment using a formerly validated NRS [29].

## Results

### Signalment

Sixteen dogs were included in the study (Golden Retriever [*n* = 4], mix breeds [*n* = 2], and one of each of the following breeds Australian Shepherd, Beagle, Boxer, Chihuahua, Cocker Spaniel, French Bulldog, Irish Soft Coated Wheaten Terrier, Lurcher, Malinois, and Yorkshire Terrier). Medical records containing age, breed, diagnosis, premedication, previous history of vestibular syndrome, and observed events of vomiting are summarised in supplementary Table 1.

Median age at onset of neurological deficits was 9.58 years (interquartile range [IQR]: 5.6–13.25 years). Ten dogs (62.5%) had no clinical signs of central involvement and symptoms were limited to the peripheral vestibular system. The majority of these dogs (7/10) showed characteristics of idiopathic vestibular syndrome, two of ten dogs were diagnosed with otitis media and interna on the affected side, and one dog showed signs of an iatrogenic peripheral vestibular syndrome after an extirpation of a trichoblastoma via bullaosteotomy. Six dogs (37.5%) showed signs consistent with central vestibular syndrome. In three out of six cases the magnetic resonance imaging (MRI) in combination with cerebrospinal fluid analysis was suggestive of neoplastic or inflammatory conditions (one dog with neoplasia, two dogs with a presumed necrotizing meningoencephalitis), whereas for the remaining three dogs no further diagnostic measures were conducted.

In the neurological examination, 14 dogs (87.5%) had head tilt and nystagmus. Ataxia was observed in 13 dogs (81.3%). All six dogs diagnosed with central vestibular syndrome demonstrated cranial nerve deficits, decreased mentation and, in all but one dog, proprioceptive deficits (Table 1).

**Table 1:** Neurological signs revealed during neurological examination

Neurological sign	Number of dogs
Head tilt	14
Nystagmus	14
Ataxia	13
Cranial nerve deficits	6
Collapse	5
Proprioceptive deficits	5
Strabismus	5
Deficits in postural reactions	4

### Behavioural assessment

**Fig. 1:**
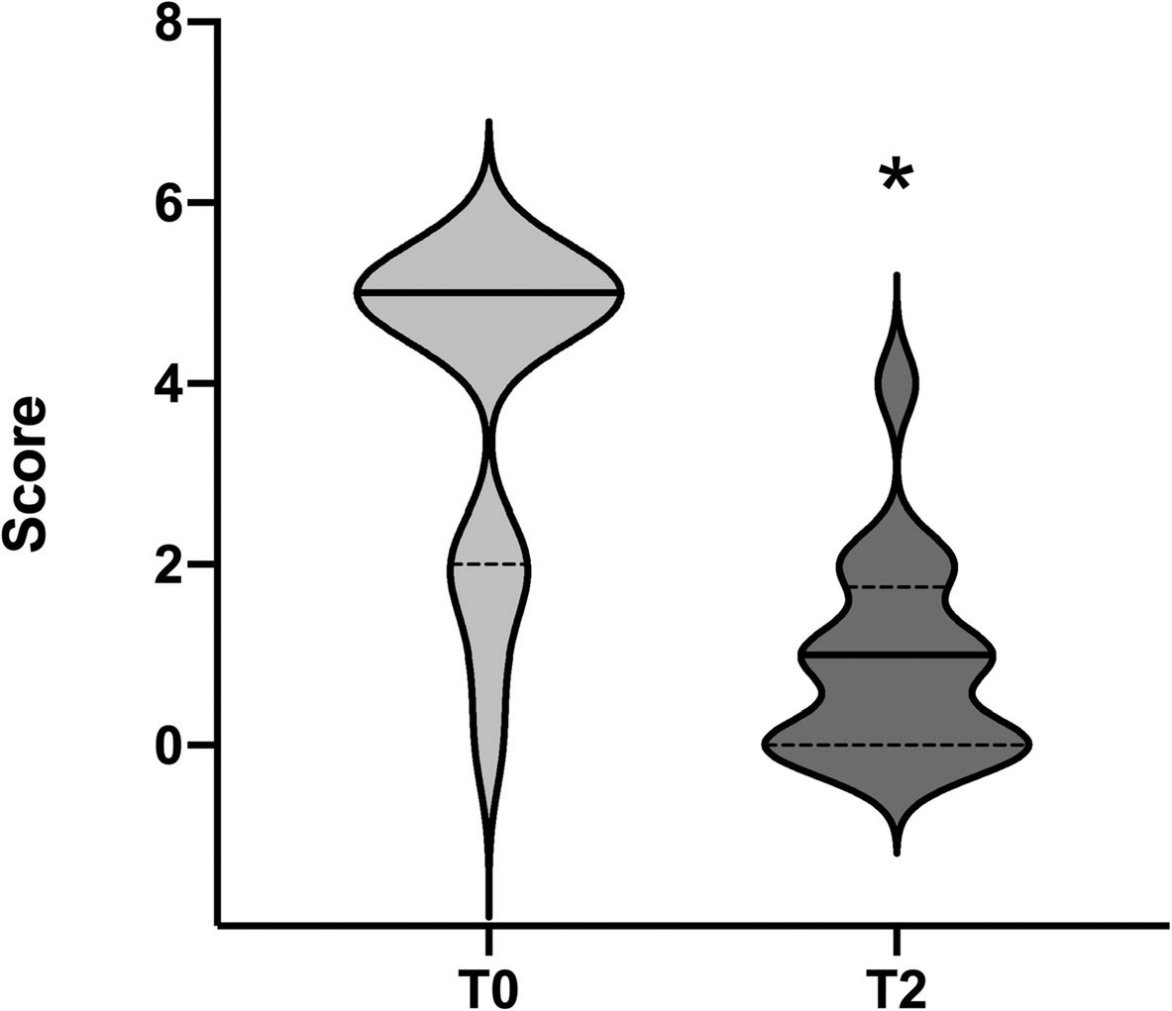
Overall nausea severity. Violin plot of NRS assessing the overall nausea severity at timepoints T0 (before ondansetron administration) and T2 (2 h after ondansetron administration) in 16 dogs with vestibular syndrome. Wilcoxon signed-rank test was used for comparison (* *p* ≤ 0.0001). Scores range from 0 to 5. See Table 2 for further information

**Fig. 2:**
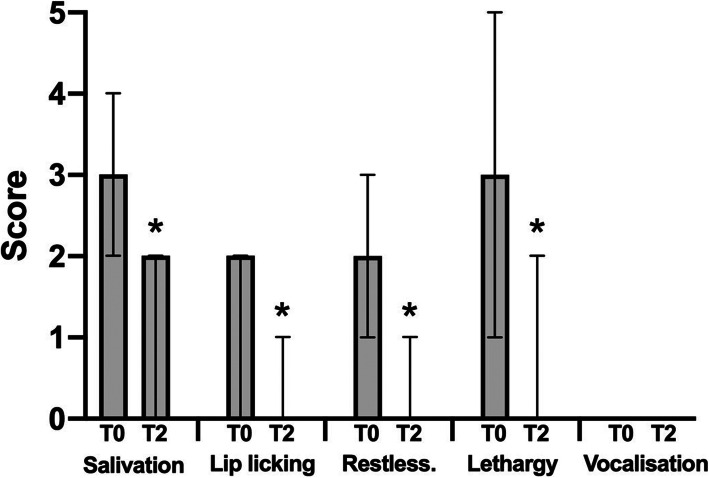
Signs indicative for nausea in the course of the study period (T0 and T2). Scores (0–5) for the five other signs indicative for nausea are shown in 11 dogs at time points T0 (before ondansetron) and T2 (2 h after ondansetron), respectively. Bars represent the median scores and error bars represent the interquartile ranges. Significant decreases of scores after ondansetron treatment are indicated by asterisks with *p* = 0.0078 for salivation, *p* = 0.0078 for lip licking, *p* = 0.0039 for restlessness, and *p* = 0.0078 for lethargy. No significant differences were found in terms of vocalisation (*p* > 0.9999) between both time points. Wilcoxon signed-rank test was used for analysis

All dogs included in the study exhibited signs of nausea. The overall severity of nausea was significantly decreased from a pre-treatment median score of 5.0 (IQR: 2.0–5.0) to post-treatment median score of 1.0 (IQR: 0–4.0, *p* ≤ 0.0001, Fig. 1). A reduction in the general nausea score was observed in all 16 dogs. The detailed assessment of each clinical sign suggestive of nausea-like behaviour (*n* = 11) revealed significant reduction in their scores from T0 to T2 for salivation (*p* = 0.0078), lip licking (*p* = 0.0078), restlessness (*p* = 0.0039), and lethargy (*p* = 0.0078) but not for vocalisation (*p* > 0.9999, Fig. 2). Vocalisation was only observed in one dog as an expression of discomfort and nausea. A total of five dogs (31.3%) showed vomitus prior to the study. The severity of nausea in dogs with vs. without vomitus (T0) was not statistically significant (*p* = 0.6223). The improvement of nausea scores in dogs with vs. without vomitus after ondansetron administration was not statistically different (*p* = 0.8587).

## Discussion

The objective of this study was to evaluate the anti-nausea efficacy of ondansetron, a 5-HT_3_ receptor antagonist, in dogs with vestibular disease. In the current open-label, prospective, multicentre study in 16 dogs with vestibular disease, the degree of nausea and clinical signs suggestive of nausea-like behaviour were significantly reduced after ondansetron administration. Around a third of the patients in this study also showed vomiting in addition to nausea, which was not observed furthermore after ondansetron was given. Despite the limitations of an open-label, prospective study, this study provides the first preliminary evidence of the potential benefit of ondansetron in the treatment of nausea induced by vestibular syndrome and highlights the importance of assessing the degree of nausea in these neurological patients.

In veterinary care, there is a need for a better awareness of veterinarians for the difference between a drug’s antiemetic and/or antinausea effect. Nausea is relatively easy to measure in people, as they can report the severity of nausea they are experiencing. In dogs, however, behaviour and facial expression must be observed precisely in order to adequately evaluate their level of nausea [8, 29]. Vomiting is often seen and described in vestibular disorders [30], however, the prevalence of preceding nausea has not been described. Radulescu et al. (2020) [2] and Schunk et al. (1988) [31] described a prevalence of vomiting of 25.7 and 40%, respectively. This is consistent with our results showing an overall prevalence of vomiting in 31.3% of dogs. Nevertheless, all dogs in our study showed clinical signs and behaviour suggestive of nausea. This supports reports that nausea and vomiting can occur separately and that it should not be assumed that a dog that is not vomiting cannot be nauseous. Studies of maropitant, an antiemetic drug developed for the use in veterinary medicine, described the cessation of vomiting but still measurable signs of nausea [11–18]. For some owners or veterinarians the absence of vomiting might be an acceptable and satisfying treatment outcome. However, human patients being treated with chemotherapeutics usually consider nausea control more important than emesis control [32, 33]. Furthermore, one could assume when nausea leads to vomiting, there is temporary relief of the nausea sensation, but blocking vomiting without relieving the nausea may be burdensome. This highlights the importance of nausea research in veterinary patients. Kraus et al. (2019) [34] also determined that many dog owners are aware of the discomfort and negative influence of nausea on animal welfare and would accept higher costs and longer hospital stays in return for an effective relief for their pets.

Golden Retriever (*n* = 4) and mixed-breed dogs (*n* = 2) were most likely to have a diagnosis of ‘vestibular syndrome’ in this case series. Bongartz et al. (2020) [35] also found mixed-breed dogs (15.1%) to be most likely affected. Similar breeds were mentioned by some recently published studies consistent with our findings [2, 36]. In the present study, the median age at onset of neurological deficits was 9.58 years. Median age reported recently elsewhere ranged from 6.8 years to 12.68 years [2, 31, 37–39]. This apparently wide range in age could represent the different study inclusion criteria. Idiopathic vestibular syndrome tends to appear more frequently in older dogs in contrast to meningoencephalitis of unknown origin, which is a frequent cause of central vestibular syndrome. It mainly affects female brachycephalic dog breeds younger than 5.5 years [39]. Neurological examination is a very accurate clinical tool to determine the presumed neuroanatomical localisation of neurological diseases within the vestibular system in more than 90% of cases, according to Bongartz et al. (2020) [35]. The main clinical findings recorded in the neurological examination of the 16 dogs were head tilt and nystagmus, followed by ataxia. These signs are the most frequently observed clinical findings consistent with vestibular syndrome [2, 31, 36, 40]. In summary, although the sample size in this case series was small, the cases included appear to be representative for the commonly affected canine population by vestibular syndrome.

The pathogenesis of nausea is complex [9, 10, 21, 22, 27, 41]. It is not completely understood and includes central and peripheral stimuli. The vestibular system involves not only the cerebellum, spinal cord, and extraocular muscles but also the cranial nerve III and the vestibular nuclei. The vestibular nuclei are directly involved in pathways for the induction of nausea in the nucleus tractus solitarius. By activation of projections to the dorsal vagal complex and ascending projections to higher brain areas like thalamus, lateral postcentral gyrus, insular cortex, and temporoparietal cortex the induction of nausea can be modulated. The complexity and multidimensional nature of nausea makes the pharmacological management challenging [33].

At receptor level, many mediators are important in the induction and modulation of the feeling of nausea, but special attention must be paid to 5-HT_3_receptors. These receptors form serotonin-gated ion channels that interact with further 5-HT receptor subtypes as well as with other neurotransmitters [24]. They have a wide distribution and range of function in the central and peripheral nervous system [42]. Neurons expressing immunoreactivity for the 5-HT_3_ receptor subtypes are located in the forebrain, brainstem (especially trigeminal motor and facial nuclei) as well as in the spinal cord [43]. In addition to other regions in the central nervous system, the distribution of 5-HT_3_ receptors in the area prostema (the chemoreceptor trigger zone), limbic and cortical regions [44], and the NTS (especially the rostral part, which interacts with higher brain regions [41]) is particularly important with regard to pharmacological antinausea intervention [45–49]. Balaban et al. (2014) [28] examined neuronal activation after galvanic vestibular stimulation in cats with the help of the detection of c-Fos expression. They concluded two networks being positively correlated with the severity of motion sickness signs. The networks include medial, lateral, superior, and inferior vestibular nuclei, the lateral nuclei of the NTS, parabrachial nucleus, and in general serotonergic and non-serotonergic projections.

Miller et al. (1996) [50] found a region in the human inferior frontal gyrus activated *via* head movement during yaw-axis rotation in the matter of number of dipoles related to the intensity of nausea. The same region was detected in functional MRI after galvanic vestibular stimulation or caloric testing. This specific region was only activated through motion sickness associated nausea and not *via* speech or finger movement, which are the normal activating stimuli of this region. The same region was also activated after the ingestion of the syrup of ipecac, a well-known herbal substance that induces nausea and vomiting. After ondansetron was applied, the intensity of dipoles in this region was significantly reduced, highlighting the strong interaction of motion sickness with nausea and ondansetron as a 5-HT_3_ receptor antagonist [41, 50]. Components of the vestibular system, representing one possible origin of nausea stimuli, have a widespread distribution of 5-HT receptors. These regions of receptor accumulation serve as possible anchor points in the axis of nausea-induction to be targeted pharmacologically.

Nausea is a subjective condition and its presence and magnitude are difficult to assess in both animals and humans, as there still is a lack of a direct measurement method until today. In humans we have to rely on self-reports while in animals we have to rely on a person’s interpretation of the observed behaviour. In human medicine, there are several types of validated scales including visual analogue scales (VAS) and numerical rating scales (NRS) in terms of self-reporting [51, 52]. Although comparing a subjective sensation between several individuals remains challenging, the recognition of nausea in humans itself is much easier than in animals. Therefore, the first step to improve the welfare of canine patients suffering from nausea is to simplify and to improve the recognition of the condition in the first place. Therefore valid methods for measurement are needed. Kenward et al. (2015) refer in their publication to pain as an analogy to nausea and compare the concept of nociception with ‘nausiception’ [8].

Both, nausea and pain, are subjective sensations which require a conscious perception of the individual and serve as a protective function. These parallels allow access to pain rating scales as possible approach to evaluate nausea, as there are no validated scores for nausea in animals. Pain has been often measured by means of simple descriptive scale (SDS), NRS or VAS with only focus on intensity [53]. Downie et al. (1978) [54] and Holton et al. (1998) [55] concluded that the NRS, which is extensively used in human medicine, represents a reliable compromise between a SDS with a lower level of sensitivity as well as the VAS and its more complicated use.

The used NRS in the present study is a modified version of the one established by Rau et al. (2010) [17] and further validated by Kenward et al. (2015) [29]. In this case series, we have chosen scale descriptions, supplementary to the one used by Kenward et al. [29], and divided them into 5 levels in accordance with the score by Rau et al. (2010) [17], relying on typical prodromal signs of nausea [10, 13, 17, 27, 28], which offers the possibility of making a better statement about the dogs’ overall level of nausea. In addition to assessment of nausea evoked behaviour patterns, the used scale also included evaluation of dog-human-interactions by evaluating lethargy and, therefore, the dogs’ reaction to external stimuli. In general, the scale descriptions facilitated and standardised evaluation although it is less sensitive than the VAS from de la Puente et al. (2007) [56] as the scores are limited to the descriptions on scale and does not allow ratings in between. De la Puente et al. (2007) [56] used a VAS that evaluated clinical signs of nausea by placing a pen mark on a 100-mm-line. The distance to the start of the line was then measured and scored in millimetres (the higher the value in mm, the stronger the clinical signs). Nevertheless, in order to minimize inconsistencies in interpretation, every dog was assessed by the same trained observer at time points before (T0) and 2 h after (T2) ondansetron administration. Another possibility for optimisation of the study would be the use of a video-based evaluation in order to exclude any influence on the observer and the canine patient. However, both observers at the RVC and the TiHo found a clinical obvious decrease in nausea-like-behaviour after ondansetron administration. The NRS with the given gradations helped to achieve a higher level of objectivity, comparability and reproducibility in this case series.

Some limitations of the present study should be noted. No blinding of observers was performed and the analyses took place at two different test centres. The observed patients in this cases series were part of a diseased population of elderly dogs (median age of onset 9.58 years) with a variety of different breeds and potentially long medical history with interfering pre-existing illnesses. These dogs were exposed to a stressful environment (hospital), where they were handled by unfamiliar people. Stress will certainly have been reflected in the dogs’ behaviour and thus may have influenced the behavioural assessment. Efforts were made in the study design to try to reduce the effect of stress by giving dogs a period of 1 h of acclimatisation. In addition to the small sample size, the absence of an untreated control group must be taken into account. As the intensity of the signs of nausea may decrease over time due to the patient’s habituation to the state of vestibular disorder, resulting in clinical improvement. As nausea varies greatly between individuals, only a placebo-controlled cross-over study can correctly compare the state of nausea in each individual. Individual adaptation to vestibular disorders is difficult to assess in clinical trials; only observation extending beyond the actual study period and assessment of effective plasma concentration can lead to preliminary evidence. However, the paired comparisons conducted between individuals showed an overall significant decrease of nausea scores in the participating dogs with every dog experiencing an improvement in the scores after ondansetron treatment.

## Conclusions

This is the first study to provide preliminary evidence of the efficacy of ondansetron as a treatment for nausea in dogs with vestibular disease. Better control of nausea will have a major impact on this patient population, as there is currently no adequate relief of nausea in veterinary medicine. Considering the limitations of this study, a placebo controlled, double-blind, randomized, cross-over study is currently under way to confirm the study results. Inclusion of the measurement of a possible objective biomarker of nausea, like arginine vasopressin, in future studies would enhance not only the interpretation of the results but also the detection of an underreported clinical sign and therefore improve canine welfare in veterinary medicine and side effect profiling in laboratory canines [8, 9, 57–59].

## Materials and methods

### Patients & inclusion criteria

Dogs were presented to the neurology and neurosurgery service at the Department of Small Animal Medicine & Surgery, University of Veterinary Medicine Hannover (TiHo), between October 2019 and March 2020 (*n* = 11) and at the Queen Mother Hospital for Animals, Royal Veterinary College (RVC), between January and August 2014 (*n* = 5). The client-owned dogs met the inclusion criteria for this open-label, multicentre pilot study if they showed clinical signs consistent with peripheral and central vestibular syndrome and nausea at the day of presentation. Owners gave informed written consent to diagnostic procedures and treatment. Medical records containing age, breed, diagnosis, premedication, previous history of vestibular syndrome, and observed events of vomiting were evaluated. Dogs presented at the RVC were given a 24 h-wash out period if they received an antiemetic drug prior to ondansetron administration in order to avoid drug interference. At the TiHo wash out periods of 15 h after maropitant (plasma concentration half-life [t_1/2_] 5.62 ± 0.77 h, post i.v. [13]) and 13 h after metoclopramide (t_1/2_ = 0.87 ± 0.17 h, post i.v. [13]) administration were provided prior to ondansetron administration. Furthermore, treatment sheets were reviewed prior to the study to avoid concomitant or prior administration of drugs with potential antinausea effects. Animal procedures undertaken were approved by the RVC’s Ethics and Welfare Committee in relation to the UK Home Office Animals (Scientific Procedures) Act 1986 (ASPA) project license 70/7269. All examinations and treatments were carried out with informed written consent of the patients‘-owners according to the ethical guidelines of the University of Veterinary Medicine Hannover.

### Study protocol

**Fig. 3:**
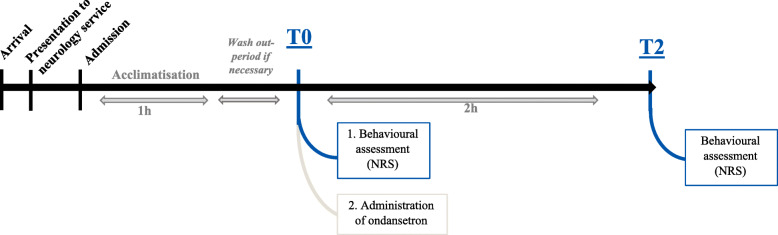
Study Timeline. After arrival at the clinic and presentation to the neurology service, the dogs were hospitalised. An acclimatisation and, if required, a wash-out period were given prior to the study start. Behavioural assessment using a Numerical Rating Scale (NRS) was conducted by a trained observer at T0 and T2. Ondansetron administration was performed straight after behavioural observations at T0

After hospitalisation, a period of at least 1 h was provided to let the dogs acclimatise to the ward. Venous access was established and all dogs were treated with fluid therapy intravenously at a maintenance dose of 2 ml/kg/h (Fig. 3). The first behavioural assessment was performed at time point T0 immediately before administration of the drug ondansetron (Cellondan®, STADAPHARM GmbH, Germany, or Zofran®, GlaxoSmithkline, UK) at a single dose of 0.5 mg/kg i.v. by the attending clinician. The ondansetron dosage used during the study corresponds to the antiemetic dose recommended by the BSAVA Small Animal Formulary [60]. Prior to application, the medication was diluted 1:1 with 0.9% saline in order to reduce the risk of phlebitis. Two hours after injection (T2), behaviour was evaluated again by the same observer.

### Behavioural assessments/nausea-score

A numerical rating scale (NRS) adapted from the 5 point-scale established by Rau et al. (2010) [17] and further developed by Kenward et al. (2015) [29] was used. One trained observer at the RVC and another at the TiHo conducted the observations in a non-blinded fashion before and after administering the drug. An assessment of general nausea-like behaviour was made on the 5-point scale, where 0 = no nausea and 5 = severe nausea, depending on the appearance and frequency of signs including salivation, lip licking, restlessness, vocalisation, and lethargy. A detailed assessment of each of these five prodromal clinical signs interpreted and suggestive for nausea-like behaviour was conducted for the eleven dogs, which presented at the TiHo (Table 2). The number of vomiting episodes was recorded for all dogs prior and during the study.

**Table 2:** Numerical rating scale (NRS). NRS scoring was used for behavioural nausea assessment at time-points T0 and T2

	Score value					
	**0 (None)**	**1 (Mild)**	**2 (Mild/Moderate)**	**3 (Moderate)**	**4 (Moderate/Severe)**	**5 (Severe)**
General nausea	No nausea	Short period of mild nausea	Longer period of mild nausea or short period of moderate nausea	Short period of moderate nausea or short period of severe nausea	Longer period of severe nausea	Constant nausea
Salivation	None	Slight dampness around the mouth	Wet around the muzzle	Pools of saliva around the lips	Dripping saliva	Strings of saliva
Lip licking	None	Occasional lip licking	Frequent lip licking	Constant lip licking for periods up to a few minutes	Frequent lip licking for periods up to several minutes	Permanent, constant lip licking
Vocalisation	None	Occasional short whining	Occasional whining	Frequent whining	Constant whining or crying for periods of a few minutes	Constant whining or crying
Restlessness	None	E.g. occasional panting/turning/circling/digging	E.g. shows longer panting/turning/circling/ digging behaviour, but calms down after a short time	E.g. anxious, repeated panting/turning/circling/digging	E.g. restless panting/turning/circling/digging behaviour, only very short calm periods between phases	E.g. does not come to rest, constant panting/turning/circling/digging
Lethargy	None	Sleeping, responsive to stimuli	Sleeping, responsive to repeated stimuli	Sleeping for long periods, responsive to stimuli	Sleeping for long periods, responsive to repeated stimuli	Sleeping for unusually long periods, unresponsive to stimuli

### Statistical analysis

All statistical analyses were performed using GraphPad Prism 9 (GraphPad Software, Inc., La Jolla, CA, USA) in order to test the hypothesis that there would be a difference in nausea-like behaviour (general nausea) and in each of the individual signs suggestive of nausea when evaluated before and after treatment with ondansetron (H1). Additionally, it was tested if dogs showing vomiting episodes had a higher degree of nausea and whether their signs of nausea, similar to those of the dogs that did not show vomiting episodes, were affected by the administration of ondansetron (H2). Data were analysed by either the Wilcoxon signed-rank test or Mann Whitney U Test, where appropriate. Data are presented as median and interquartile range. Tests were two-tailed and *p* ≤ 0.05 was considered statistically significant.

## Supplementary Information

**Additional file 1.** Medical records of the included dogs with vestibular syndrome.

## Data Availability

The datasets used and/or analysed during the current study are available from the corresponding author on reasonable request.
