# Supplementary material for: The use of ondansetron for the treatment of nausea in dogs with vestibular syndrome
Source: BMC Vet Res. 2021 Jun 21;17:222. doi: 10.1186/s12917-021-02931-9 (PMC8218477; doi:10.1186/s12917-021-02931-9)
Supplement: Supplementary file 1 — Additional file 1. Medical records of the included dogs with vestibular syndrome. [file 12917_2021_2931_MOESM1_ESM.pdf]

Supplementary table 1: Medical records of all 16 dogs with vestibular syndrome and associated nausea.

Study ID	Centre	Breed	Age in months	Clinical signs	History of vestibular syndrome	History of vomiting	Medication history	Neuroanatomical localization	Disease
**1**	RVC	Golden Retriever	116	Ataxia, collapse, head tilt, nystagmus	No	No	Carprofen (within previous 24h)	Peripheral	Idiopathic vestibular syndrome
**2**	RVC	Cocker Spaniel	168	Ataxia, head tilt	No	Yes	Dexamethasone (48h prior to study), maropitant (24h prior to study)	Peripheral	Idiopathic vestibular syndrome
**3**	RVC	Lurcher	114	Collapse, head tilt, nystagmus	No	Yes	Maropitant (24h prior to study)	Peripheral	Idiopathic vestibular syndrome
**4**	RVC	Golden Retriever	160	Collapse, head tilt, nystagmus	Yes	No	Maropitant (24h prior to study), meloxicam, compund sodium lactate fluids (60ml/h)	Peripheral	Idiopathic vestibular syndrome
**5**	RVC	Yorkshire Terrier	80	Ataxia, head tilt	No	No	None	Peripheral	Idiopathic vestibular syndrome
**6**	TiHo	Irish Soft Coated Wheaten Terrier	94	Ataxia, head tilt, nystagmus	No	Yes	Carprofen, maropitant (17 h prior to study)	Peripheral	Iatrogenic (after extirpation of a trichoplastoma via bullaosteotomy)
**7**	TiHo	Crossbreed	136	Ataxia, collapse, cranial nerve deficits, nystagmus, pendular head movement, proprioceptive deficits	No	No	MCP (13 h prior to study)	Central	Neoplasia
**8**	TiHo	French Bulldog	37	Ataxia, head tilt, nystagmus	No	No	Phenylbutazon + prednisolon	Peripheral	Otitis media et interna
**9**	TiHo	Boxer	83	Ataxia, facial nerve paresis, head tilt, nystagmus, strabismus	No	Yes	None	Peripheral	Otitis media et interna

**10**	TiHo	Chihuahua	58	Ataxia, cranial nerve deficits, head tilt, nystagmus, postural reactions , proprioceptive deficits, strabismus	No	No	Clindamycin, maropitant (15 h prior to study)	Central	Necrotizing meningoencephalitis (NME)
**11**	TiHo	Golden Retriever	162	Ataxia, cranial nerve deficits, head tilt, nystagmus, postural reactions , proprioceptive deficits, strabismus	No	No	Maropitant (17 h prior to study)	Central	Unknown
**12**	TiHo	Crossbreed	29	Ataxia, cranial nerve deficits, head tilt, nystagmus	No	No	Antibiotics (unknown), cortisone, metamizol	Central	Necrotizing meningoencephalitis (NME)
**13**	TiHo	Golden Retriever	159	Ataxia, head tilt, nystagmus	No	No	None	Peripheral	Idiopathic vestibular syndrome
**14**	TiHo	Malinois	64	Collapse, , nystagmus, strabismus, proprioceptive deficits, postural reactions	No	No	None	Central	Unknown
**15**	TiHo	Australian Shepherd	155	Ataxia, head tilt, nystagmus, strabismus	No	No	None	Peripheral	Idiopathic vestibular syndrome
**16**	TiHo	Beagel	157	Ataxia, cranial nerve deficits, head tilt, nystagmus, proprioceptive deficits, postural reactions	No	Yes	Imepitoin	Central	Unknown
